# Clinical outcomes of HER2-positive metastatic breast cancer patients with brain metastasis treated with lapatinib and capecitabine: an open-label expanded access study in Korea

**DOI:** 10.1186/1471-2407-12-322

**Published:** 2012-07-28

**Authors:** Jungsil Ro, Sohee Park, Sung- Bae Kim, Tae You Kim, Young Hyuk Im, Sun Young Rha, Joo Seop Chung, Hanlim Moon, Sergio Santillana

**Affiliations:** 1 National Cancer Center Hospital and Research Institute, Goyang, Gyeonggi-do, 410-769, Republic of Korea; 2 Department of Oncology, Asan Medical Center, University of Ulsan College of Medicine, Seoul, Republic of Korea; 3 Department of Internal Medicine, Seoul National University Hospital, Seoul, Republic of Korea; 4 Department of Hematology-Oncology, Samsung Medical Center, Seoul, Republic of Korea; 5 Department of Internal Medicine, Severance Hospital, Seodaemun-gu, South Korea; 6 Deparment of Medical Oncology, Pusan National University Hospital, Busan, Republic of Korea; 7 Department of Medical (AP Oncology). GlaxoSmithKline Oncology, Seoul, Republic of Korea

**Keywords:** Brain Metastasis, HER2-positive Metastatic Breast Cancer, Lapatinib and Capecitabine Therapy, LEAP

## Abstract

**Background:**

To evaluate efficacy in patients with brain metastasis (BM) on entry into the lapatinib expanded access program (LEAP).

**Methods:**

LEAP is a worldwide, single-arm, open-label study. HER2-positive, locally-advanced or metastatic breast cancer patients with progression after an anthracycline, taxane, and trastuzumab were eligible. Patients received capecitabine 2000 mg/m^2^ daily in two divided doses, days 1–14, every 21 days and lapatinib 1250 mg once daily.

**Results:**

Among 186 patients enrolled in 6 Korean centers, 58 had BM. Progression-free survival (PFS) was 18.7 weeks in patients with BM and 19.4 weeks without BM (P = 0.88). In patients with BM, brain response was synchronized with systemic responses (P = 0.0001). Overall survival (OS) was 48.9 weeks in patients with BM and 64.6 weeks without BM (P = 0.23). Multivariable analysis found hormone receptor positivity (P = 0.003) and clinical benefit rate (CBR) of combined systemic and brain disease (P < 0.0001) significantly associated with prolonged brain PFS, and CBR of combined systemic and brain disease (P = 0.03) and longer trastuzumab use (P = 0.047) associated with prolonged OS in patients with BM; prior capecitabine did not affect PFS or OS in patients with BM.

**Conclusion:**

Lapatinib plus capecitabine is equally effective in patients with or without BM.

**Trial registration:**

ClinicalTrials.gov (NCT00338247)

## Background

Patients with HER2-positive breast cancer who have been treated with trastuzumab are at greater risk for developing brain metastasis, with the incidence ranging from 25% to 36% [1-4]. Therefore, anti-HER2 therapy that has central nervous system (CNS) activity may decrease its incidence or benefit these patients as a salvage therapy.

Lapatinib is an oral small molecule tyrosine kinase inhibitor that targets epidermal growth factor receptors 1 and 2 (HER2). Lapatinib offers a treatment option for HER2-positive patients who progress on trastuzumab and has been shown to reduce the risk of disease progression when combined with capecitabine, paclitaxel, or letrozole in patients with HER2-positive MBC [5-7]. The registration trial was terminated prematurely after demonstrating a significant benefit in time to progression (TTP) for lapatinib plus capecitabine versus capecitabine alone in women with MBC that had been previously treated with an anthracycline, a taxane, and trastuzumab (hazard ratio [HR] = 0.51; 95% confidence interval [CI], 0.35 to 0.74; P < 0.001) [5]. An exploratory analysis of data from the trial showed that fewer patients in the lapatinib plus capecitabine arm developed new brain metastases compared to capecitabine alone (2% vs 6%; P = 0.045) [5,8]. As a small molecule, lapatinib may cross the blood-brain barrier and has shown evidence of CNS activity based on preclinical and clinical evidence [9-11].

In order to accommodate patient demand for lapatinib after the positive results from the registration trial, two expanded access studies were initiated: Lapatinib Expanded Access Program (LEAP) and French Authorisation Temporaire d’Utilisation (ATU) [12-14]. Certain patients were included in LEAP that would have been excluded from the registration study, including patients with symptomatic brain metastases and those with prior capecitabine exposure [5]. Therefore, data from LEAP augments the information available from the registration trial.

The current report focuses on clinical efficacy outcomes data from Korean patients with brain metastasis who were treated in LEAP.

## Methods

Full details of the LEAP study design have been previously described [12]. Briefly, LEAP is a single-arm, open-label, expanded access study that enrolled patients worldwide, including 6 centers in Korea. Enrollment in Korea commenced January 2007 and concluded April 2008.

Patients aged ≥ 18 years with HER2-positive, locally advanced or metastatic breast cancer were eligible if their cancer had progressed after treatment with an anthracycline, a taxane, and trastuzumab given alone or in combination in either the metastatic or adjuvant setting. HER2 status was assessed locally. Additionally patients with non-measurable disease, ECOG performance status 2, and prior capecitabine therapy were included. Patients with CNS metastases were eligible if steroid requirement was minimal regardless of CNS symptoms.

Patients received capecitabine 2000 mg/m^2^ per day in two divided doses, days 1 to 14, every 21 days and lapatinib 1250 mg once daily. Physicians could delay or adjust dosages of either medication for associated toxicities. Efficacy assessments occurred at 6-week intervals. Response and disease progression were investigator assessed according to modified Response Evaluation Criteria in Solid Tumors (RECIST version 1.0) criteria [15]. For brain metastasis, magnetic resonance imaging (MRI) reports by the participating centers were retrospectively interpreted as follows: complete response (CR) when the tumor disappeared completely; partial response (PR) when the tumor shrank by more than 30% of the longest diameter; any response when the reports described any degree of response or decreased size without mentioning tumor dimension; stable disease (SD) when the tumor did not change in size; and progressive disease (PD) when the reports described any degree of increase.

Analyses for progression-free survival (PFS) and overall survival (OS) were performed using Kaplan-Meier survival analysis. Progression-free survival is calculated from the administration date of study drug until PD or death from any cause. An exploratory analysis for patients with brain metastasis on study entry was performed using the same methods. Brain PFS was analyzed and calculated from the beginning of study drug to the date of PD in the metastatic brain tumors. Multivariable analyses were conducted using Cox’s proportional hazards model.

Each patient provided written informed consent and participating institutional review boards approved the study. Financial support was provided by GlaxoSmithKline. The study is registered at ClinicalTrials.gov (NCT00338247).

## Results

### Patients with brain metastasis

Of a total 186 patients, 58 had brain metastasis on entry. All patients but one had HER2-positive breast cancer defined as 3+ immunohistochemistry or gene amplification by fluorescence in situ hybridization. Twenty eight patients (48.3%) were estrogen receptor (ER) and/or progesterone receptor (PR) positive. Baseline characteristics are shown in Table 1. Compared to patients without brain metastasis, those with brain metastasis had a longer median duration of prior trastuzumab therapy (35.4 weeks vs 20.6 weeks; P = 0.02). Prior to LEAP enrollment, local treatment of brain metastasis included whole brain radiation (n = 35), stereotactic radiosurgery or gamma-knife surgery (n = 10), surgical excision (n = 3), and intrathecal therapy for leptomeningeal disease (n = 1). Four patients underwent more than one modality and six patients did not receive local CNS therapy. The median duration of study drug treatment was similar between patients with and without brain metastasis (18.6 weeks vs 19.1 weeks, respectively; P = 0.78).

**Table 1:** Baseline patient characteristics

	**bb Lapatinib + Capecitabine**											
**Characteristic**	**Brain metastasis (n = 58)**		**No Brain metastasis (n = 128)**		***P*value**^**a**^	**No Prior capecitabine (n = 95)**		**Prior capecitabine (n = 91)**		***P*value**^**a**^	**Total (n = 186†)**	
	**No.**	**%**	**No.**	**%**		**No**.	**%**	**No.**	**%**		**No.**	**%**
**Median age, yrs (range)**	46.5	(27-70)	49	(27-71)	0.170	48	(27-71)	47	(29-70)	0.960	48	(27-71)
<50	38	(65.5)	67	(52.3)	0.1111	53	(55.8)	52	(57.1)	0.8832	105	(56.2)
≥50	20	(34.5)	61	(47.7)		42	(44.2)	39	(42.9)		82	(43.9)
**Hormone receptor**												
ER + and/or PR+	28	(49.1)	45	(35.4)	0.103	36	(38.3)	37	(41.1)	0.764	73	(39.7)
ER- and PR-	29	(50.9)	82	(64.6)		58	(61.7)	53	(58.9)		111	(60.3)
*Unknown*	*1*	*(1.7)*	*1*	*(0.8)*		*1*	*(1.0)*	*1*	*(1.1)*		*2*	*(1.1)*
**No. metastatic sites**												
1	5	(8.9)	17	(13.6)	0.174	14	(15.2)	8	(9.0)	0.042	22	(12.2)
2	15	(26.8)	47	(37.6)		37	(40.2)	25	(28.1)		62	(34.3)
≥ 3	36	(64.3)	61	(48.8)		41	(44.6)	56	(62.9)		97	(53.6)
*Unknown*	*2*	*(3.4)*	*3*	*(2.3)*		*3*	*(3.2)*	*2*	*(2.2)*		*5*	*(2.7)*
**Pattern of metastasis**												
Visceral only	18	(31.6)	56	(45.5)	0.161	34	(37.8)	40	(44.4)	0.617	74	(41.1)
Non-visceral only	8	(14.0)	11	(8.9)		11	(12.2)	8	(8.9)		19	(10.6)
Both	31	(54.4)	56	(45.5)		45	(50.0)	42	(46.7)		87	(48.3)
*Unknown*	*1*	*(1.7)*	*5*	*(3.9)*		*5*	*(5.3)*	*1*	*(1.1)*		*6*	*(3.2)*
**Prior capecitabine**^**b**^	30	(51.7)	61	(47.7)		0	(0.0)	91	(48.9)		91	(48.9)
**> 3 Prior chemotherapy regimens**	22	(37.9)	45	(35.2)		10	(10.5)	57	(62.6)		67	(36.0)
**Median duration of trastuzumab, wks (range)**	35.4	(4.0-113.6)	20.6	(0.1-105.0)	0.016	27.3	(0.1-104.4)	21.6	(3.0-113.6)	0.252	24.1	(0.1-113.6)
**Interval from last dose of trastuzumab prior to study entry, wks**												
Median (range)	11.6	(2.7-146.6)	20.9	(0.1-172.3)	0.365	7.7	(0.1-172.3)	24.9	(1.4-160.9)	0.001	14.6	(0.1-172.3)
< 4	3	(5.2)	16	(12.6)	0.341	14	(14.7)	5	(5.6)	<0.001	19	(10.3)
4 - 8	17	(29.3)	33	(26.0)		37	(39.0)	13	(14.4)		50	(27.0)
>8	38	(65.5)	78	(61.4)		44	(46.3)	72	(80.0)		116	(62.7)
*Unknown*	*0*	*(0.0)*	*1*	*(0.8)*		*0*	*(0.0)*	*1*	*(1.1)*		*1*	*(0.5)*

^a^ Fisher’s exact χ2 test for categorical variables and independent *t-*test for continuous variables; two-sided *p*-values.

^b^ Included 8 cases who had lapatinib during capecitabine.

^†^ Because of few information (only age and study date), 1 patient was excluded.

‘Unknown’ included missing values.

### Efficacy

While central review of MRI was not feasible, we construed response from the serial MRI reports, which were read by independent radiologists from each center.

Five of 58 patients did not have repeat brain MRIs ever or upon ending study drugs, including one consent withdrawal. In an additional patient, brain MRI was taken long after ending study drugs. Another patient achieved systemic PR but the treating physician did not repeat brain MRI until brain symptoms occurred after ending study drugs. There were 4 patients who entered study after complete surgical excision of BM (n = 3) and gamma knife surgery (n = 1). Because of the possibility that these 4 patients (Table 2, Case No.: 8–11) with no evidence of disease may have artificially influenced the brain PFS, we checked for the heterogeneity in brain PFS among patients grouped by their treatment before entering the study (no local treatment vs surgical or local radiotherapy vs whole brain radiotherapy) and found that the difference was not statistically significant (log-rank test, P = 0.6281) (Table 3). These four patients remained without evidence of disease in the brain on study drugs (n = 1, Case No 10) or beyond the end of study drugs (n = 3, Case No, 8,9,11) with a median brain PFS of 31.4 (range, 21.6–34.7) weeks.

**Table 2:** Characteristics of 11 patients with not evaluable or uncertain brain response

**Case No**	**Study drug started**	**Study drug ended**	**Reason for ending drugs**	**Best systemic response**	**Brain PD by symptoms and MRI**	**Reason for not evaluable brain response during study period**	Inclusion*
1	Consent withdrawal						●
2	2007.3.22	2007.3.29	Unknown	Unknown	Unknown	No follow-up MRI	●
3	2008.2.19	2008.3.11	Unknown	Unknown	Unknown	No follow-up MRI	●
4	2008.2.15	2008.4.1	Systemic PD	PD	Unknown	No follow-up MRI	●
5	2007.3.22	2007.4.17	Systemic PD	PD	Unknown	No follow-up MRI	●
6	2007.8.07	2007.9.17	Systemic PD	PD	2008.2.18	MRI long after ending study drug	●, ◊
7	2007.4.02	2008.5.21	Brain PD	PR	2008.6.3	No MRI until brain symptoms	●, ◊
8	2007.3.30	2007.6.28	Systemic PD	PR	2007.11.12	Metastatectomy prior to study started	◊
9	2007.11.30	2008.2.5	Systemic PD	SD < 6mo	2008.7.30	Metastatectomy prior to study started	◊
10	2008.4.8	2008.11.11	Systemic PD	SD ≥ 6mo	2008.11.7	Metastatectomy prior to study started	◊
11	2007.9.5	2007.12.31	Systemic PD	SD < 6mo	2008.2.3	Gamma knife surgery prior to study started	◊

* Included denominator for ●, brain response rate; ◊, brain PFS analysis.

These 11 patients excluded the association between brain response and systemic response (Table 3).

**Table 3:** Brain PFS Heterogeneity by Treatment Effects using Log-rank test

**Best brain response**	**CR**		**PR**		**Any Response**		**SD ≥ 6mo**		**SD < 6mo**		**PD**		**Total**		**Log-rank test for treatment,*P*-value**
**Treatment before entering the study**	**No.**	**Median brain PFS, wk (95 % CI)**	**No.**	**Median brain PFS, wk (95 % CI)**	**No.**	**Median brain PFS, wk (95 % CI)**	**No.**	**Median brain PFS, wk (95 % CI)**	**No.**	**Median brain PFS, wk (95 % CI)**	**No.**	**Median brain PFS, wk (95 % CI)**	**No.**	**Median brain PFS, wk (95 % CI)**	
No	0	NE	2	59.3 (NE)	0	NE	1	30.4 (NE)	1	12.6 (NE)	2	9.2 (6.4-12.0)	6	21.5 (12.0-59.3)	0.6281
Excision/GKS/ SRS/FSRT	4	32.4 (21.6-34.7)	0	NE	6	27.5 (20.0-53.7)	2	36.4 (28.1-44.7)	0	NE	0	NE	12	31.4 (22.1-34.7)	
WBRT	2	84.3 (NE)	4	21.1 (15.9-NE)	13	40.9 (25.0-59.0)	5	35.0 (26.1-NE)	3	17.9 (12.6-24.0)	5	10.7 (4.0-32.6)	32	31.1 (23.7-41.0)	
Subtotal	6	33.6 (30.4-84.3)	6	42.4 (16.6-NE)	19	32.9 (23.7-54.1)	8	33.2 (28.1-48.6)	4	15.2 (12.6-24.0)	7	10.7 (6.4-12.1)	50	30.6 (24.0-34.7)	
IT MTX					1	17.0 (NE)							1	17.0 (NE)	
Total	6	33.6 (30.4-84.3)	6	42.4 (16.6-NE)	20	32.8 (22.1-54.1)	8	33.2 (28.1-48.6)	4	15.2 (12.6-24.0)	7	10.7 (6.4-12.1)	51	30.4 (23.7-34.7)	

CR, complete response; FSRT, fractionated stereotactic radiotherapy; GKS, gamma knife surgery; IT MTX, intrathecal methotrexate; PR, partial response; PD, progressive disease; SD, stable disease; SRS, stereotactic radio-surgery; WBRT, whole-brain radiotherapy; NE, not estimable.

Thus, 11 patients were excluded in the analysis of brain response, but were included in the overall prognosis from systemic disease and 6 of these patients were included in brain PFS (Table 2).

Among 47 patients evaluable and 7 patients (Table 2 Case No 1–7) not evaluable for brain response, 2 patients achieved CR, 6 patients achieved PR, and 20 patients experienced some degree of radiologic improvement. Two patients with CR received prior whole brain radiation. One of these patients showed residual brain tumors on entry but achieved a CR, documented even in the last MRI taken a long time after study drug ended, whose study drugs were discontinued because of systemic PD. The other patient who experienced brain PD during whole brain radiation, achieved and remained in CR in both systemic and brain disease until the last follow-up date on study drugs. We added 7 non-evaluable patients in the denominator for tumor response to lessen the selection bias on the basis of mere availability of follow-up MRIs. Thus 28 of 54 patients (51.9%, 95% CI: 38.5–65.2) experienced disease remission or any degree of tumor shrinkage on LEAP. Stable disease ≥ 6 months was observed in an additional 8 patients and SD < 6 months was observed in 4 patients; 7 patients developed PD.

To assess the association between the brain and systemic responses, the 11 patients listed in the Table 2 with non-evaluable or uncertain brain response were excluded. There was a significant association between the brain and systemic responses in patients with BM (P = 0.0001) (Table 4).

**Table 4:** Association between brain response and systemic response in patients with brain metastasis at study entry

**Best Systemic Response**	**Best Brain Response**														
	**CR**		**PR**		**Any response**		**SD > 6 m**		**SD < 6 m**		**PD**		**Total**		*P*-value*
	**No**	**(%)**	**No**	**(%)**	**No**	**(%)**	**No**	**(%)**	**No**	**(%)**	**No**	**(%)**	**No**	**(%)**	
**CR**	0	(0.0)	0	(0.0)	0	(0.0)	0	(0.0)	0	(0.0)	0	(0.0)	0	(0.0)	0.0001
**PR**	1	(50.0)	2	(33.3)	12	(60.0)	2	(25.0)	0	(0.0)	1	(14.3)	18	(38.3)	
**SD > 6 m**	0	(0.0)	0	(0.0)	3	(15.0)	6	(75.0)	1	(25.0)	0	(0.0)	10	(21.3)	
**SD < 6 m**	1	(50.0)	4	(66.7)	3	(15.0)	0	(0.0)	3	(75.0)	2	(28.6)	13	(27.7)	
**PD**	0	(0.0)	0	(0.0)	2	(10.0)	0	(0.0)	0	(0.0)	4	(57.1)	6	(12.8)	
**Total**	2	(3.7)	6	(11.1)	20	(37.0)	8	(14.8)	4	(7.4)	7	(13.0)	47	(100.0)	

* The patients with evaluable response were calculated by Fisher’s exact test.

CR, complete response; PR, partial response; SD, stable disease; PD, progressive disease.

**Figure 1:**
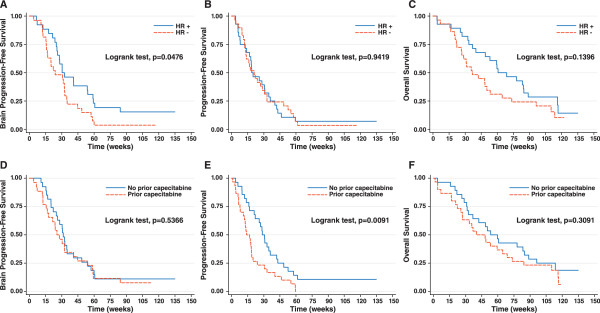
Kaplan-Meier estimates for (A) brain PFS by hormone receptor (HR) status; (B) PFS by hormone receptor (HR) status; (C) OS by hormone receptor (HR) status; (D) brain PFS by prior capecitabine; (E) PFS by prior capecitabine; and (F) OS by prior capecitabine in patients with brain metastasis on entry.

While overall PFS in patients with BM was not statistically significantly different based on hormone receptor subsets (HR = 1.02, 95% CI: 0.60-1.74; P = 0.94), brain PFS was shorter in patients with hormone receptor- negative disease (HR = 1.78, 95% CI: 1.00-3.19; P = 0.05; Figure 1).

In the overall population (n = 186), median PFS was 18.7 weeks in patients with BM and 19.4 weeks in patients without BM (HR 0.98 [95% CI: 0.71-1.35], P = 0.88). Among patients with BM, median overall PFS was significantly prolonged in patients who did not use prior capecitabine (28.3 weeks vs 14.0 weeks, P = 0.009), but brain PFS was not statistically significantly different between these two subsets (31.4 weeks vs 25.6 weeks, P = 0.54; Figure 1).

Combined brain and systemic responses were a significant factor for brain PFS (HR = 3.65, 95% CI: 1.95-6.85; P < 0.0001) and OS (HR = 2.18, 95% CI: 1.07-4.42; P = 0.031) in both univariate and multivariable analyses (Table 5).

**Table 5:** Univariate and multivariable analyses of brain PFS and OS in patients with brain metastasis at baseline (N = 53)

**Risk Factor**	**Univariate analysis**						**Multivariable analysis**^**a**^					
	**Brain PFS**	**OS**	**Brain PFS**	**OS**								
	**HR**	**95 % CI**	***P***	**HR**	**95 % CI**	***P***	**HR**	**95 % CI**	***P***	**HR**	**95 % CI**	***P***
Prior capecitabine *vs.* no prior capecitabine	1.20	0.68-2.11	0.5377	1.22	0.67-2.22	0.5195						
Hormone receptor positive *vs.* hormone receptor negative	0.56	0.31 -1.00	0.0507	0.62	0.34-1.13	0.1190	0.38	0.20-0.72	0.0031			
Best systemic response SD < 6 m, PD *vs.* CR, PR, SD ≥ 6 m	2.47	1.39-4.40	0.0022	3.55	1.89-6.67	<0.0001						
Median trastuzumab duration, wks ≥35.4 *vs.* <35.4	0.85	0.48-1.50	0.5672	0.45	0.24-0.85	0.0140				0.50	0.25-0.99	0.0476
Age at ≥50 *vs.* <50	0.67	0.36-1.23	0.1925	1.13	0.61-2.13	0.6958						
No. of metastasis ≥3 *vs.* <3	0.81	0.44-1.48	0.4918	1.10	0.57-2.13	0.7733						
No. of prior chemotherapy regimens >3 *vs.* ≤ 3	0.88	0.48-1.61	0.6835	1.48	0.79-2.76	0.2169						
Pattern of metastasis Visceral only vs other	1.51	0.80-2.85	0.1994	1.16	0.58-2.31	0.6778						
Median TTbrainP, wks ≥30.7 *vs.* <30.7				0.44	0.24-0.82	0.0095				0.58	0.29-1.16	0.1223
Median interval from brain metastasis diagnosis to study enrollment, wks ≥8.1 *vs.* <8.1	1.42	0.80-2.53	0.2365	1.49	0.81-2.74	0.2032						
Brain response* SD < 6 m, PD *vs.* CR, PR, any response, SD ≥ 6 m	7.44	3.42-16.18	<0.0001	1.79	0.85-3.77	0.1263						
Brain response & systemic response Else *vs .* Both of CR, PR, SD ≥ 6 m	2.82	1.57-5.09	0.0006	3.16	1.68-5.96	0.0004	3.65	1.95-6.85	<0.0001	2.18	1.07-4.42	0.0311

^a^ Risk factors were determined using univariate Cox’s proportional hazards regression (α <0.10).

* excluded 2patients with not evaluable reponse.

CR, complete response; PR, partial response; SD, stable disease; PD, progressive disease; TTbrainP, time to brain progression.

HR, hazard ratio; CI, confidence interval.

Overall survival among patients with BM was prolonged in patients who used trastuzumab longer in the past (median duration of ≥35.4 weeks,

HR = 0.50, 95% CI: 0.25-0.99; P = 0.047). Of patients with BM, those with hormone receptor-positive disease lived longer (median 68.7 weeks vs 42.3 weeks), but the difference did not reach statistical significance (P = 0.14; Figure 1).

Reasons for stopping study drugs among patients with BM were brain PD in 16 patients (29%), systemic PD in 20 patients (36%), both brain and systemic PD in 15 patients (27%) and adverse events in 1 patient (2%). Three patients (5%) continue to take both study drugs on study as of the cut-off date, May 20, 2010.

### Overall population

Baseline characteristics for the overall population are shown in Table 1. Two patients with HER2-negative or unknown status were included in the study. Patients with prior capecitabine harbored a greater number of metastatic sites (P = 0.04). The proportion of patients with ≥ 3 metastatic sites was 62.9% among those with prior capecitabine compared to 44.6% among those without prior capecitabine. The median interval between the last dose of trastuzumab and study entry was longer among patients with prior capecitabine exposure (24.9 weeks vs 7.7 weeks; P = 0.001). The median duration of study drug treatment for all patients was 19 weeks (range, 0.14-146.9+) with a significantly longer duration in patients with no prior capecitabine (median 24 weeks vs 17.9 weeks; P = 0.01).

### Efficacy

In all patients, the overall response rate was 37.7%, with a median PFS of 19.4 weeks (95% CI = 18.3-24.0) and median OS of 59.4 weeks (95% CI = 49.7-68.3). New brain metastases developed in 8 patients (6.2%) among the 128 patients without brain metastasis at baseline during the study. Brain was the only site of disease progression in six of these patients.

**Figure 2:**
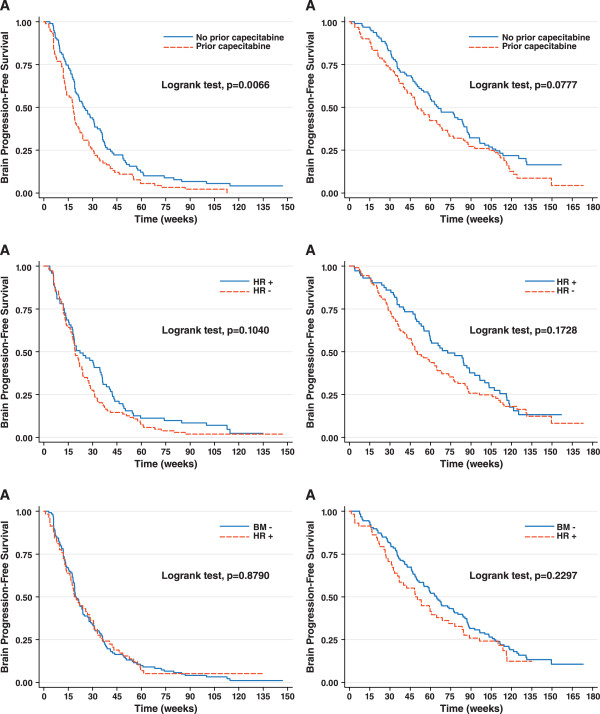
In the overall population regardless of brain metastasis status, Kaplan-Meier estimates for (A) PFS by prior capecitabine; (B) OS by prior capecitabine; (C) PFS by hormone receptor (HR) status; (D) OS by HR status; (E) PFS by brain metastasis status in; (F) OS by brain metastasis status

A statistically significant prolongation in PFS was observed in patients with no prior capecitabine exposure compared to those with exposure (HR = 1.50, 95% CI: 1.12-2. 02; P = 0.006; Figure 2A). Median PFS was 25.0 weeks in patients without prior capecitabine exposure and 18.0 weeks in patients with exposure. Consistent with these observations, overall response rates were lower among patients with prior capecitabine exposure (data not shown). A trend toward prolonged OS in patients without prior capecitabine was apparent but not statistically significant (HR = 1.33 [95% CI, 0.97-1.83]; P = 0.08; Figure 2B). Between hormone receptor-positive and hormone receptor-negative patients in the overall population, hormone receptor-positive patients lived longer, but no statistical significance was observed in PFS (HR = 0.78 [95% CI, 0.57-1.06]; P = 0.10; Figure 2C) or OS (HR = 0.80 [95% CI, 0.57-1.11]; P = 0.17; Figure 2D).

Multivariable analysis failed to reveal a significant benefit associated with no prior capecitabine for PFS or OS in the overall population (Table 6). Variables significantly associated with prolonged PFS were non-visceral metastasis, longer prior trastuzumab use, and systemic response to study drugs. Significant variables for prolonged OS were fewer metastatic sites, fewer prior chemotherapy regimens, longer trastuzumab use, and longer time to progression.

**Table 6:** Univariate and multivariable analysis of PFS and OS in all patients (N = 186)

**Risk Factor**	**Univariate analysis**						**Multivariable analysis**^**a**^					
	**PFS**	**OS**	**PFS**	**OS**								
	**HR**	**95 % CI**	***P***	**HR**	**95 % CI**	***P***	**HR**	**95 % CI**	***P***	**HR**	**95 % CI**	***P***
Prior capecitabine *vs.* no prior capecitabine	1.50	1.12-2.02	0.0072	1.33	0.97-1.83	0.0790	1.22	0.89-1.67	0.2140	0.73	0.48-1.10	0.1351
Hormone receptor positive *vs.* hormone receptor negative	0.78	0.57-1.06	0.1064	0.80	0.57-1.11	0.1741						
Best systemic response SD, PD *vs.* CR, PR	2.54	1.85-3.48	<0.0001	1.88	1.33-2.64	0.0003	2.60	1.87-3.63	<0.0001	1.31	0.86-1.99	0.2064
Median prior trastuzumab duration, wks ≥24.1 *vs.* <24.1	0.68	0.50-0.91	0.0108	0.57	0.41-0.78	0.0006	0.66	0.48-0.89	0.0074	0.68	0.48-0.96	0.0271
Age ≥50 *vs.* <50	0.89	0.66-1.20	0.4418	0.84	0.61-1.15	0.2726						
No. of metastasis ≥3 *vs.* <3	1.16	0.86-1.16	0.3427	1.64	1.18-2.28	0.0034				1.78	1.23-2.58	0.0023
No. of prior chemotherapy regimens >3 *vs.* ≤3	1.17	0.86-1.58	0.3214	1.58	1.14-2.19	0.0067				1.64	1.08-2.47	0.0194
Pattern of metastasis Visceral only *vs.* other	1.58	1.17-2.15	0.0031	0.98	0.70-1.36	0.8928	1.61	1.18-2.21	0.0031			
Median TTP, wks ≥20.0 *vs.* <20.0				0.36	0.26-0.50	<0.0001				0.37	0.25-0.56	<0.0001
Brain metastasis *vs.* no brain metastasis	0.98	0.71-1.35	0.8979	1.23	0.88-1.74	0.2310						

^a^ Risk factors were determined using univariate Cox’s proportional hazards regression (α <0.10).

CR, complete response; PR, partial response; SD, stable disease; PD, progressive disease; TTP, time to disease progression.

HR, hazard ratio; CI, confidence interval.

No significant difference was observed between patients with or without BM in PFS (HR = 0.98; P = 0.88; Figure 2E) or OS (HR 1.23; P = 0.23; Figure 2F). As of the data cut-off date, 48 of 58 patients with brain metastasis (83%) and 105 of 128 patients without brain metastasis (82%) had died.

## Discussion

The LEAP study offered the opportunity to evaluate patients with brain metastases and those with prior capecitabine exposure. Data from this study provided evidence that patients with HER2-positive brain metastasis achieved a significant clinical benefit from receiving lapatinib plus capecitabine therapy. Patients who entered the study with brain metastasis had similar PFS to those without brain metastasis (median 18.7 weeks vs 19.4 weeks, respectively; P = 0.88) but shorter OS (median 48.9 vs 64.6, respectively; P = 0.23) without statistical significance. The similar PFS reflected the efficacy of lapatinib in combination with capecitabine in the intracranial metastatic lesions. These data are supported by the phase II study results by Lin, et al showing that lapatinib with capecitabine has activity in CNS metastasis [11]. Among 242 patients with brain metastasis in their series, CNS objective responses to lapatinib were observed in 6% of patients. However in an exploratory analysis, 21% of patients experienced a ≥20% volumetric reduction in their CNS lesions. Of the 50 evaluable patients who entered the lapatinib plus capecitabine extension portion of that study, 20% experienced a CNS objective response and 40% experienced a ≥20%volumetric reduction in their CNS lesions. Although they appear similar in response rates between the studies by extrapolation, we are aware that response evaluation may not be the optimal method to assess the potential anticancer activity in brain tumor lesions as pointed out by Therasse, et al in the RECIST guidelines [15]. In such cases, PFS can be considered a valuable alternative to evaluate clinical efficacy [15].

Furthermore, we examined whether the objective responses and/or decreases in tumor size in the CNS paralleled those of the extra-CNS disease. Indeed, the data showed that those who responded in the brain tended to have systemic response with statistical significance (P = 0.0001).

A retrospective analysis of 126 metastatic breast cancer patients with brain metastasis revealed prolonged survival associated with hormone receptor-positive compared to hormone receptor-negative subtype in HER2-positive disease when measured from the date of systemic recurrence (median, 27.4 months vs 20.9 months) or from brain metastasis (median, 9.2 months vs 5.0 months) [16]. Other studies have indicated a poorer prognosis associated with hormone receptor-negative subtype compared to hormone receptor-positive in patients with HER2-positive MBC not necessarily harboring brain metastasis [17]. The current study corroborates these data with a statistically significant prolonged brain PFS in patients with hormone receptor-positive/HER2-positive breast cancer compared to the hormone receptor-negative/HER2-positive subtype. Overall survival was prolonged as well (HR = 1.61) but did not reach statistical significance in this small population (P = 0.12).

Unlike the lapatinib pivotal trial [5], this study provided an opportunity to evaluate outcomes in patients according to prior capecitabine exposure. Although patients with brain metastasis without prior capecitabine exposure achieved longer overall PFS (median; 28.6 weeks vs 14 weeks, P = 0.009), there was no difference in brain PFS regardless of prior capecitabine use. The majority of the patients without brain metastasis who had history of disease progression on prior capecitabine experienced clinical benefit by the reuse of capecitabine in combination with lapatinib to a certain extent (median TTP 26.9 weeks without prior capecitabine vs 18.0 weeks with prior capecitabine; P = 0.002 ). Moreover, prior capecitabine was not statistically significantly associated with OS in multivariate analysis. These results are consistent with the analysis of the worldwide LEAP population, in which median PFS was 23.9 weeks in patients without prior capecitabine exposure and 18.4 weeks in patients with prior exposure [12]. This clinical benefit by capecitabine retrial in combination with lapatinib could be partly explained by the in vitro synergistic cytotoxicity between a lapatinib analog and the capecitabine metabolite 5'-deoxy-5-fluorouridine against breast cancer cell lines [18]. Other possible confounding factors related with the poorer outcomes in association with prior capecitabine therapy were a higher rate of patients who had received >3 prior chemotherapy regimens. At baseline, patients with prior capecitabine had a higher number of metastatic sites, an additional finding that supports more advanced disease in this group.

Efficacy outcomes in the overall Korean population (median PFS, 20 weeks; median survival, 60 weeks) are consistent with experience with lapatinib plus capecitabine in the worldwide LEAP population (median PFS, 21 weeks; median OS, 40 weeks) [12]. Although the study is limited by a non-randomized design, and a retrospective evaluation of brain metastasis, this study provides data on a broader population than was tested in the lapatinib registration trial and offers specific efficacy information in this selected population who harbor brain metastasis.

## Conclusions

In patients with HER2-positive brain metastasis who received lapatinib plus capecitabine, combined brain and systemic responders and patients with hormone receptor-positive disease achieved prolonged PFS and those who achieved both brain and systemic responses and used prior trastuzumab for longer experienced prolonged survival. Additionally, the combination of lapatinib plus capecitabine in the overall population was equally effective in patients with or without brain metastasis.

## Competing interests

The following authors of this paper declare that there is no conflict of interest involved in this paper: SP, SBK, TYK, YHI, SYR, JSC. The following authors declare a conflict of interest: JR received honoraria from Glaxosmithkline for an advisory board meeting, HM and SS are both employed by Glaxosmithkline and own Glaxosmithkline stock.

## Authors’ contributions

JR, SP conceived and designed the study, assembled/analyzed data, and prepared the manuscript, SBK, TYK, YHI, SYR, JSC, participated in the study design, data collection, assembly/analysis of data, and manuscript writing. HM, SS participated in the study design, assembly/analysis of data, and manuscript writing. All authors read and approved the final manuscript. Ro J, Park S, Kim S B, et al. Clinical outcomes of brain metastasis by lapatinib (L) and capecitabine (C) in an open-label expanded access study among Korean patients with HER2 positive metastatic breast cancer. Abstract and poster P1-14-04.

## Pre-publication history

The pre-publication history for this paper can be accessed here:

http://www.biomedcentral.com/1471-2407/12/322/prepub
